# Exploiting ChatGPT for Diagnosing Autism-Associated Language Disorders and Identifying Distinct Features

**DOI:** 10.21203/rs.3.rs-4359726/v1

**Published:** 2024-05-21

**Authors:** Chuanbo Hu, Wenqi Li, Mindi Ruan, Xiangxu Yu, Lynn K. Paul, Shuo Wang, Xin Li

**Affiliations:** 1 Department of Computer Science, University at Albany, Albany, 12222, NY, USA.; 2 Humanities and Social Sciences, California Institute of Technology, Pasadena, 91125, CA, USA.; 3 Department of Radiology, Washington University in St. Louis, St. Louis, 63110, MO, USA.; 4 Lane Department of Computer Science and Electrical Engineering, West Virginia University, Morgantown, 26506, WV, USA.

**Keywords:** Autism spectrum disorder, Language deficits, Machine learning, Large language models, ChatGPT

## Abstract

Diagnosing language disorders associated with autism is a complex and nuanced challenge, often hindered by the subjective nature and variability of traditional assessment methods. Traditional diagnostic methods not only require intensive human effort but also often result in delayed interventions due to their lack of speed and specificity. In this study, we explored the application of ChatGPT, a state-of-the-art large language model, to overcome these obstacles by enhancing diagnostic accuracy and profiling specific linguistic features indicative of autism. Leveraging ChatGPT’s advanced natural language processing capabilities, this research aims to streamline and refine the diagnostic process. Specifically, we compared ChatGPT’s performance with that of conventional supervised learning models, including BERT, a model acclaimed for its effectiveness in various natural language processing tasks. We showed that ChatGPT substantially outperformed these models, achieving over 13% improvement in both accuracy and F1-score in a zero-shot learning configuration. This marked enhancement highlights the model’s potential as a superior tool for neurological diagnostics. Additionally, we identified ten distinct features of autism-associated language disorders that vary significantly across different experimental scenarios. These features, which included echolalia, pronoun reversal, and atypical language usage, were crucial for accurately diagnosing ASD and customizing treatment plans. Together, our findings advocate for adopting sophisticated AI tools like ChatGPT in clinical settings to assess and diagnose developmental disorders. Our approach not only promises greater diagnostic precision but also aligns with the goals of personalized medicine, potentially transforming the evaluation landscape for autism and similar neurological conditions.

## 1 Introduction

Autism spectrum disorder (ASD) is a developmental condition characterized by challenges in social interaction, restricted interests, and repetitive behaviors [1–3]. The spectrum of ASD symptoms is broad, with communication difficulties often standing out as the most significant and impacting aspects of the disorder [4–6]. These symptoms manifest differently across various age groups. In adults, communication difficulties are particularly pronounced and can significantly impact social integration and personal development[7, 8]. Properly identifying and understanding these communication issues in adults is crucial for effective intervention and support.

Language disorders in adults with ASD include a wide range of issues, from the absence of speech to subtle impairments such as echolalia (repetitive use of phrases or sounds), pronoun reversal, and pragmatic language impairments [9–11]. These disorders can hinder effective communication and pose significant challenges in social and occupational settings. Accurate and early identification of these language anomalies is essential for providing appropriate therapeutic interventions that can significantly enhance the quality of life for adults with ASD.

While the Autism Diagnostic Observation Schedule, Second Edition (ADOS-2) [12] is a gold standard for ASD diagnosis across various age groups, its application, especially in adults, can be limited by the subjective interpretations required of human clinicians. Traditional methods are not only time-consuming but also prone to inconsistencies due to varied human judgment, which can affect the reliability of diagnosing complex language and social communication issues in adults.

Large language models (LLMs) have the potential to transform the practice of precision medicine [13] and personalized healthcare [14]. For example, LLMs can analyze vast amounts of medical literature and patient data to provide personalized medical advice. Considering an individual’s medical history, genetic information, and lifestyle, these models can suggest tailored health recommendations, treatments, and preventive measures. Meanwhile, LLMs can serve as advanced decision-support tools for healthcare providers by offering up-to-date medical information, diagnostic suggestions, and treatment options based on the latest research findings. This support is crucial in handling complex cases such as autism spectrum disorder (ASD) where multiple disorders or conditions may affect the patient.

Connecting LLMs with ASD is still an emerging field that has not attracted much attention as of today for the following reasons. First, popular LLMs such as BERT [15] and ChatGPT [16] are trained on general text repositories without any domain knowledge injected. It requires fine-tuning LLMs on ASD-related datasets to leverage their power for supporting the diagnosis and intervention of ASD. Second, ASD is characterized by sophisticated social communication and interaction behaviors, including language skills. However, language skills alone are insufficient for a reliable diagnosis of ASD. How to combine language disorders in ASD with other salient biomarkers (e.g., gaze and stimming related [17]) remains an under-researched topic. Third, unlike other neurological disorders such as depression and anxiety, ASD is a developmental disorder, which make its data collection challenging. In addition to privacy concerns, ASD can be confused with the delay in language development among young children [18]. In other words, the potential use of LLMs under the ASD context has to properly take inevitable bias into account [19].

In this study, we employed machine learning and LLMs to investigate language deficits in ASD by the following motivations:

- **Advancement in Diagnostic Accuracy and Efficiency.** The diagnosis of language disorders associated with autism presents numerous challenges, including variability in symptom presentation and overlap with other developmental issues. Traditional diagnostic processes are often lengthy, subjective, and require extensive human effort, which may delay intervention. Utilizing ChatGPT, a state-of-the-art large language model, can enhance diagnostic accuracy and efficiency. This AI-powered approach can process natural language inputs at scale and identify nuanced patterns that human evaluators might miss. By integrating ChatGPT, clinicians can rapidly analyze language use patterns, thereby speeding up the diagnostic process and enabling earlier therapeutic interventions.
- **Identification of Specific Linguistic Features.** Current methods for identifying language disorders in autistic individuals primarily rely on general assessments that may not capture all the specific features relevant to the disorder. ChatGPT’s capabilities include deep learning algorithms that excel in pattern recognition across large datasets. By exploiting these capabilities, the study aims to refine the profiling of language disorders by pinpointing distinct linguistic features most indicative of autism. These features include, but are not limited to, echolalia, pronoun reversal, and atypical use of language functions, which are critical for tailoring personalized treatment plans.
- **Contribution to Personalized Medicine.** Personalized medicine in ASD treatment plans is pivotal for effective intervention. By accurately diagnosing and specifically identifying the features of language disorders with ChatGPT, practitioners can better understand their patients’ individual needs. This targeted approach not only improves outcomes but also aids in the development of personalized educational and therapeutic strategies. Furthermore, this research could pave the way for creating more sophisticated AI tools that can be adapted to other cognitive and developmental disorders, potentially revolutionizing the field of neurodevelopmental diagnostics.

## 2 Results

### 2.1 ChatGPT Model for ADOS Audio Dataset

We established the following computational framework:

#### Dataset.

For the purpose of this study, we utilized the Caltech ADOS Audio Dataset, which comprises audio recordings from diagnostic interviews conducted under the ADOS-2, Module 4. This dataset includes recordings from 44 subjects diagnosed with ASD. Each subject participated in 15 different scenarios that are designed to elicit social and communicative behaviors characteristic of individuals on the autism spectrum. Out of these scenarios, we specifically selected 11 that focus on social language interactions between the examiner and the patient. These selected scenarios provide a concentrated dataset to analyze social communicative exchanges, crucial for identifying language-related symptoms of ASD.

#### A4 Score and Labeling.

The A4 score is a critical metric from the ADOS-2, Module 4, which assesses the ”Stereotyped/Idiosyncratic Use of Words or Phrases.”In our dataset: thirteen subjects had a score of 0, indicating minimal or no use of stereotyped language. Twenty-seven subjects had a score of 1, showing mild repetitive or formal use of language that is not obviously odd. Four subjects had a score of 2, frequently using stereotyped or odd phrases. For the purposes of this research, we combined the subjects with scores 1 and 2 into a single category (Category 1). Those with a score of 0 were grouped into Category 0. This categorization allowed us to simplify the binary classification task.

#### Evaluation Metrics.

The effectiveness of the ChatGPT model was evaluated using several metrics: Accuracy: The proportion of total diagnoses that were correctly identified by the model. Precision: The ratio of correct positive observations to the total predicted positives. Recall (Sensitivity): The ratio of correct positive observations to the actual positives in the data. F1 Score: The harmonic mean of precision and recall, providing a single metric to assess the balance between precision and recall.

### 2.2 Comparison of Language Deficit Diagnosis

#### 2.2.1 Evaluation of LLM Performance

In evaluating the effectiveness of various large language models for diagnosing language disorders associated with autism, we performed a comparative analysis focusing on several key performance metrics: accuracy, precision, recall, and F1 score. The models compared include XLNet, ALBERT, DistilBERT, RoBERTa, BERT, and our ChatGPT-based approach. Table 2 summarizes the results of this comparison.

Our ChatGPT-based model demonstrated superior performance across all metrics when compared to the other models. Specifically, ChatGPT achieved an accuracy of 81.82%, a precision of 82.45%, a recall of 81.82%, and an F1 score of 79.89%. This represents a significant improvement over the highest performing baseline model, BERT, which scored 63.92% in accuracy and 61.87% in F1 score. Notably, the improvements in accuracy and F1 score by our model were over 12% and 18% better than the BERT model, respectively. Therefore, the substantial gains in performance metrics underscore the effectiveness of the ChatGPT model in handling the nuances of language processing related to ASD. Accurately diagnosing SLD linked to ASD is essential for early intervention and effective treatment planning.

#### 2.2.2 Evaluation of Speaker Diarization

To further examine the impact of utilizing speaker diarization, we conducted ablation experiments that varied the use of speaker diarization tools integrated with our ChatGPT-based approach:

The integration of Google’s speaker diarization (SD) technology with our ChatGPT-based model (w/Google) markedly enhanced all performance metrics, achieving the highest scores across the board. Unlike other diarization tools, Google SD not only distinguishes multiple speakers (e.g., Speaker 1, Speaker 2) but crucially identifies whether the speaker is the examiner or the patient. This capability is particularly beneficial for downstream tasks where understanding the interaction dynamics and the role of each speaker (examiner vs. patient) significantly influences the model’s performance in contextual analysis and response generation.

Together, these results validate the potential of incorporating sophisticated AI-driven tools like speaker diarization with ChatGPT to enhance the accuracy and efficiency of diagnostics.

### 2.3 Analysis of Features of Language Deficit

#### 2.3.1 Correlation Analysis

**Fig. 1:**
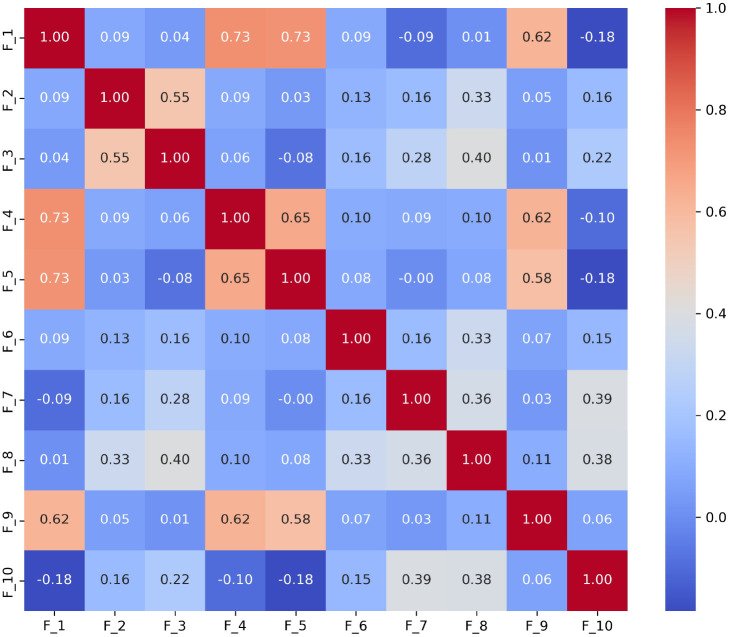
Correlation Coefficients Between Features of Language Deficit

To thoroughly understand the interrelationships between different language features identified in the ASD diagnostic assessments, We analyzed the interrelationships among ten language features (*F*_1_ to *F*_10_) derived from the Caltech dataset. These features represent various aspects of language use that may indicate ASD, such as repetitive use of words or unusual language patterns. We calculated Pearson correlation coefficients between each pair of features to quantify their linear relationships. Each language feature is represented as a binary variable, where ‘1’ indicates the presence and ‘0’ indicates the absence of that specific language disorder feature within any given sample. For example, if a feature detected by ChatGPT such as ”echolalic repetition” is observed in the dialogue during a diagnostic session, it is marked as ‘1’ for that session; otherwise, it is marked as ‘0’. This binary coding allows us to apply Pearson correlation to measure the linear relationship between each pair of features across all samples. This analysis helps in pinpointing which features tend to co-occur within the linguistic profiles of ASD diagnosed through ADOS-2, Module 4. The computed correlation matrix for the features is presented in Figure 1. Detailed observations from Figure 1 are as follows:

##### 1. Highly Correlated Features:

- *F*_1_, *F*_4_, and *F*_5_: These features show very high correlations (*r* = 0.734 between *F*_1_ and *F*_4_, *r* = 0.727 between *F*_1_ and *F*_*5*_, and *r* = 0.655 between *F*_4_ and *F*_5_). This suggests they may capture similar aspects of linguistic behavior, possibly related to the repetitive or stereotyped use of language, which is a common indicator of ASD.
- *F*_4_ and *F*_9_: Another pair, *F*_4_ and *F*_9_ (*r* = 0.622), indicates a strong association, which might reflect overlapping features of language presentation in ASD, such as idiosyncratic language use or atypical language processing.

##### 2. Moderately Correlated Features:

- *F*_2_ and *F*_3_, *F*_8_ and *F*_7_: These features exhibit moderate correlations (*r* = 0.549 for *F*_2_ and *F*_3_, *r* = 0.363 for *F*_8_ and *F*_7_) that are significant but lower than those of *F*_1_, *F*_4_, and *F*_5_. They likely indicate a less direct but still meaningful relationship in linguistic traits, such as variability in speech that includes both repetitive and novel elements.

##### 3. Negatively Correlated Features:

- *F*_1_ and *F*_10_: The negative correlation (*r* = −0.184) suggests that when *F*_1_ (possibly denoting less severe ASD indicators) is present, *F*_10_ (perhaps denoting more severe ASD indicators) is less likely to be present, and vice versa. This can help differentiate levels of language impairment in ASD diagnoses.

Together, the exploration of the correlation between features of language deficit in ASD offers valuable insights into the complex nature of communication challenges faced by individuals on the spectrum.

#### 2.3.2 Distribution of Features of Language Deficit Across Scenarios

This subsection analyzes the correlations between linguistic features across various ADOS scenarios to identify patterns that may indicate language disorders associated with ASD. The focus is on scenarios that involve direct dialogue between the examiner and the patient, reflecting our study’s emphasis on communicative interactions. While Scenarios *S*_1_, *S*_2_, *S*_8_, and *S*_10_ provide valuable insights into various aspects of cognitive and social functioning, they were not included in this analysis due to their lack of direct dialogue-based interaction between the examiner and the patient, which is a primary focus of our research. To effectively analyze the differences in the distribution of values from F_1_ to *F*_10_ across various scenarios, we conducted a detailed statistical examination (see Table 3). This analysis helps to understand how the prevalence of each linguistic feature associated with ASD varies across the scenarios, which can provide insights into the contexts or conditions under which certain features are more likely to appear.

We have derived the following observations and insights:

- **Feature Prevalence:** The occurrence rates of features *F*_2_, *F*_6_, and *F*_7_, which represent aspects of unconventional content, verbal fluency, and excessive social phrasing respectively, were consistently above 60% across most scenarios. This high prevalence underscores their significance as key indicators of ASD.
- **Language Feature in Social Contexts:** Features such as *F*_1_ (possibly related to echolalia or repetitive speech), *F*_4_, *F*_5_, and *F*_9_ (potentially related to atypical or stereotyped language use) were entirely absent in several scenarios, underscoring their sensitivity to specific social or communicative contexts.
- **Scenario-Specific Patterns:** High prevalence rates in *F*_7_ during the *S*_7_ (i.e., ‘Emotions’) scenario, and diverse responses in *F*_2_ across the *S*_12_ (i.e., ‘Friends, Relationships, and Marriage’) and *S*_15_ (i.e., ‘Creating a Story’) scenarios suggest that certain linguistic features were particularly elicited by emotional or social relational contexts.

**Fig. 2:**
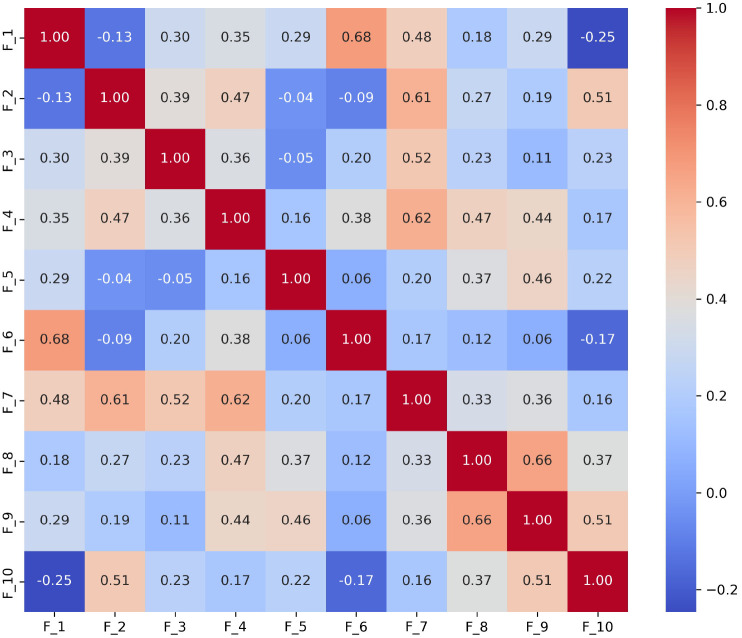
Correlation matrix of linguistic features *F*_1_ to *F*_10_ in the *S*_3_ (i.e., ‘Description of a Picture’) scenario. The matrix shows strong correlations between features, underscoring the interdependencies that influence how visual information is described.

**Fig. 3:**
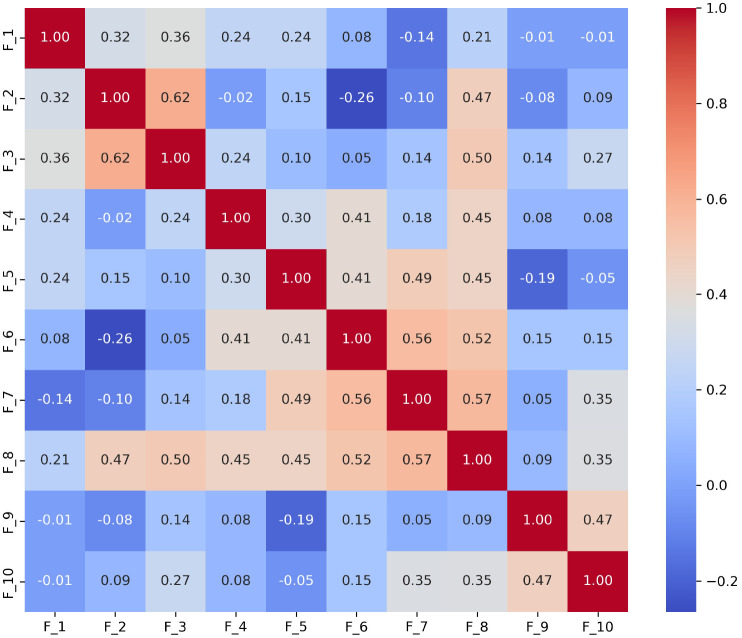
Correlation matrix of linguistic features *F*_1_ to *F*_10_ in the *S*_9_ (i.e., ‘Cartoons’) scenario. This matrix highlights correlations that elucidate the cognitive and perceptual challenges in interpreting cartoons, essential for understanding narrative contexts and humor in ASD.

To further demonstrate the utility of this analysis, we specifically focused on the *S*_3_ and *S*_9_ scenarios, which are essential for evaluating narrative skills and abstract reasoning, respectively. We derived the following results:

- **Scenario** *S*_3_ (Figure 2): The strong correlation between *F*_1_ and *F*_6_ (0.68) indicates challenges in effectively summarizing visual content. This may reflect difficulties in processing and conveying information succinctly, which is often a challenge for individuals with ASD. A high correlations (0.66) between *F*_8_ (monotone social expression) and *F*_9_ (stereotyped media quoting) suggests that individuals may struggle with varying their emotional expressions, which could affect the emotional richness of their speech. Correlations between *F*_7_ (excessive social phrasing) and *F*_4_ (incongruous humor) (0.62), and between *F*_7_ and *F*_2_ unconventional content (0.61) suggest a connection between repetitive social expressions and the production of either inappropriate humor or atypical content. This pattern may indicate that individuals with ASD use scripted language as a strategy to manage social interactions, although this can often result in conversations that seem awkward or misplaced.
- **Scenario *S***_**9**_ (Figure 3): The strong correlation between *F*_2_ and *F*_3_ (0.62) suggest that individuals with ASD might struggle to adjust their language to fit the context appropriately. This is particularly problematic in scenarios like watching and discussing cartoons, where understanding shifting dialogues and multiple characters’ perspectives is essential; Additionally, *F*_8_’s significant correlations with *F*_7_ (0.57) reflects difficulties in varying emotional tone and using phrases that might be socially appropriate. Individuals exhibiting these features tend to speak in a flat, unmodulated manner while possibly overusing certain social phrases, making their speech seem rigid and scripted. Such speech patterns can make it difficult for them to engage in spontaneous and emotionally responsive interactions, which are critical for successful social exchanges.

Together, these correlations suggest co-morbid linguistic challenges that individuals with ASD may encounter in scenarios requiring detailed visual interpretation or complex narrative understanding.

### 2.4 Case Study

Lastly, we present two case studies to illustrate the practical application of ChatGPT in identifying language deficits in ASD.

Table 4 and 5 show the dialogue between an examiner (E) and a patient (P). It showcases typical conversational challenges faced by individuals with ASD. The patient’s responses highlight several linguistic features that indicate underlying language disorders.

Together, these case studies demonstrate the effectiveness of using ChatGPT, combined with structured conversational analysis, to diagnose social language disorders in ASD. The identified features align well with known ASD communication challenges, providing a robust basis for further diagnostic evaluation and intervention planning.

## 3 Discussion

This study leveraged the ChatGPT model augmented with Google’s speaker diarization and transcription technologies to analyze language patterns in the Caltech ADOS Audio Dataset. The dataset included audio recordings from 44 adults diagnosed with ASD, focusing on scenarios that elicit social language interactions critical for diagnosing language-related symptoms of ASD. The incorporation of ChatGPT significantly enhanced the diagnostic process, yielding superior performance metrics compared to other models like BERT [15], RoBERTa [23], and XLNet [20], particularly in terms of accuracy, precision, recall, and F1 score.

One of the most significant outcomes of this study is the improved capability of ChatGPT to recognize and understand nuanced language deficits, which are often subtle and complex in adults with ASD. The model’s advanced analytical abilities allowed for a deeper examination of speech patterns and linguistic anomalies that traditional methods might miss. Our study complements the recent study of large language models (LLM) for encoding clinical knowledge [24]. Note that our current understanding of ASD from a clinical perspective is still limited. We expect that LLM such as ChatGPT might help enrich or expand clinical knowledge from clinical data in the future.

This enhanced detection is crucial, as it enables clinicians to diagnose more accurately and also contributes to a better understanding of the linguistic challenges faced by individuals with ASD. Our effort is well aligned with the existing project of building language resources for ASD [25]. Particularly, the use of Google’s speaker diarization technology has proven essential in accurately identifying the speaker roles (examiner vs. patient), which significantly impacts the model’s ability to analyze dialogue effectively. This synergy not only enhances diagnostic accuracy but also enriches the data quality with well-attributed, contextually segmented transcripts, facilitating more detailed and precise language analysis.

The results from the present study have strong clinical implications. Our model’s ability to dissect complex language interactions and identify nuanced language use offers a profound advantage in clinical settings. It allows for an earlier and more accurate detection of language deficits, which are often indicative of ASD. This early diagnosis is crucial for the timely intervention that can lead to better management and outcomes for adults with ASD. A promising future direction is to leverage the power of LLM into distinguishing ASD from other language impairment during the development [26]. If LLM can shed new insight to the co-occurrence of ASD and language impairment, clinical diagnosis of developmental disorders might benefit from human-AI collaboration.

Despite its innovations, this study has limitations that should be addressed in future research. The effectiveness of the ChatGPT model depends heavily on the quality and variety of the training data. The current dataset, while substantial, represents a relatively homogeneous population in terms of linguistic and cultural backgrounds. Expanding the dataset to include a more diverse population could help improve the model’s robustness and generalizability. Furthermore, future studies could explore the integration of multimodal data analysis [27] to enhance diagnostic capabilities further. Combining speech with visual cues such as facial expressions and body language could provide a more comprehensive view of an individual’s communicative and social behaviors. Additionally, refining the models to incorporate feedback loops that allow continual learning from new data can adaptively improve their diagnostic accuracy over time [28].

## 4 Methods

### 4.1 Evaluating Autism in Adults: The ADOS-2 Module 4 Diagnostic Process

The ADOS-2 [29, 30] is an update and extension of the original ADOS, which is a standardized diagnostic tool for ASD. The ADOS assesses communication, social interaction, play, and restricted and repetitive behaviors. It provides a series of structured and semi-structured tasks that involve social interactions between the examiner and the person being assessed. Module 4 of the ADOS-2 is designed for verbally fluent adolescents and adults (see Table 6 for description of tasks). In addition, Module 4 of the ADOS-2 organizes observations into five main areas, assessing various aspects of interaction and communication critical for diagnosing ASD in verbally fluent adolescents and adults. Table 7 provides a summary of these categories, including the specific items they encompass and their respective descriptions: each participating in 15 different scenarios (see Table 6) designed to elicit communicative and social responses that are indicators of ASD. The scenarios were structured to cover a comprehensive range of social interactions and communicative behaviors.

In language-based diagnostics, the **A4 score** — part of the Stereotyped Behaviors and Restricted Interests category — becomes particularly relevant. This score assesses:

- **0** = ”Rarely or never uses stereotyped or idiosyncratic words or phrases.”
- **1** = ”Use of words or phrases tends to be more repetitive or formal than that of most individuals at the same level of expressive language, but not obviously odd, OR occasional stereotyped utterances or odd use of words or phrases, with substantial spontaneous, flexible language as well.”
- **2** = ”Often uses stereotyped utterances or odd words or phrases, with some other language.”
- **3** = ”Frequently uses odd or stereotyped speech, and rarely uses non-stereotyped spontaneous speech.”

The A4 score assesses the use of stereotyped language, which is a critical indicator of ASD. A higher A4 score suggests a more frequent use of stereotyped or idiosyncratic speech, aiding in the diagnosis of ASD with higher specificity and sensitivity.

### 4.2 Framework for Diagnosing Autism and Identifying Language Disorders

**Fig. 4:**
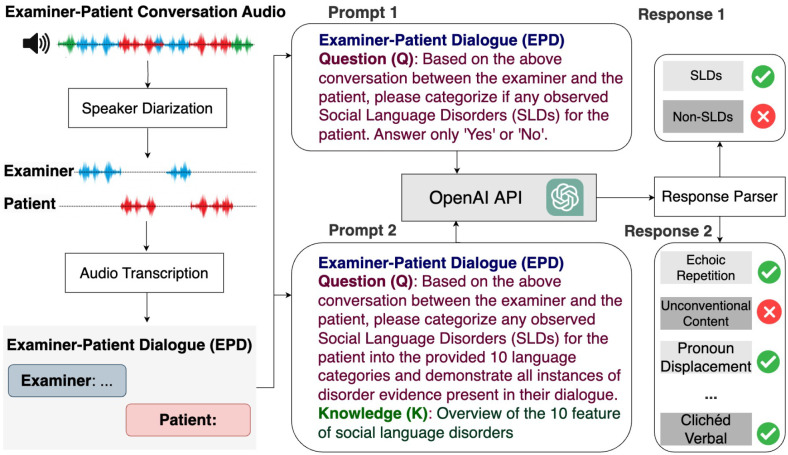
Framework for Diagnosing Autism and Identifying Language Disorders

Building on the foundational practices established by the ADOS-2, specifically Module 4 designed for verbally fluent adolescents and adults, we have developed a comprehensive framework (see Figure 4) that incorporates LLMs like ChatGPT. This framework is tailored to enhance the diagnostic precision and identification of language disorders in individuals suspected of having ASD. Specifically, it involves the following components:

- **Speaker Diarization and Audio Transcription.** This technology segments the audio recordings to precisely separate the speech of the examiner from that of the patient. Such separation is crucial as it enhances the understanding of the patient’s behavior in conversational contexts by isolating their verbal responses, which are then analyzed for potential linguistic abnormalities. The audio segments identified through diarization are subsequently transcribed into text using Google’s state-of-the-art transcription technologies (see Table 4). This conversion facilitates a detailed examination of the social language used by the patient, aiding in the detection of disorder-specific features within their speech.
- **Language Pattern Analysis Using ChatGPT.** In this framework, ChatGPT is utilized not only to diagnose ASD but also to identify specific language disorder characteristics that are indicative of ASD. The process begins with the preparation of structured prompts that are designed to elicit comprehensive information from the dialogues between examiners and patients. These prompts are crafted as follows:
  - **Examiner-Patient Dialogue (EPD):** The dialogue text, which includes conversational exchanges between the examiner and the patient, serves as the primary input for ChatGPT. This dialogue is carefully processed to maintain the integrity and context of the interaction, ensuring that all relevant linguistic cues are preserved.
  - **Question Design:** To guide the analysis, specific questions are formulated based on the dialogue content. These questions aim to direct ChatGPT’s attention to potential signs of language disorders, such as repetitive phrasing, atypical language use, or disrupted conversational flow.
  - **Knowledge Design:** This component incorporates domain-specific knowledge from autism diagnostics, which is used to refine ChatGPT’s responses. By integrating expert knowledge, the model is better equipped to recognize and interpret the subtle nuances that characterize ASD-related language disorders.
  - **Prompt Integration:** The complete prompt for ChatGPT includes the dialogue text, the targeted questions, and the expert knowledge cues. This integrated approach helps in precisely pinpointing disorder characteristics that might be overlooked in a less structured analysis.
- **Functionality of the Response Parser**: The response parser is a critical element that processes the outputs from ChatGPT. It performs two main functions:
  - **Diagnosis of Language Disorders**: It evaluates ChatGPT’s responses to identify language disorders in each scenario task. If a disorder is present in any task, the subject is predicted to have a language disorder associated with ASD. It categorizes these disorders based on predefined criteria that reflect typical ASD manifestations in language.
  - **Identification of Specific Characteristics**: Beyond mere diagnosis, the response parser also identifies specific characteristics of the language disorders. It extracts detailed information about the nature and extent of the linguistic anomalies detected, such as the type of stereotypy or idiosyncrasy in the patient’s speech.

### 4.3 Examiner-Patient Audio Separation Based on Speaker Diarization

This subsection details the utilization of Google’s advanced audio analysis tools to implement speaker diarization and audio transcription, facilitating the effective separation of examiner and patient speech in audio recordings. This technology is pivotal for analyzing communicative interactions in our study, which focuses on identifying language disorders associated with ASD.

Speaker diarization is the process of partitioning an audio stream into homogeneous segments according to the identity of the speaker. It involves distinguishing speakers in an audio recording and attributing speech segments to those individual speakers without prior knowledge of their identities. This process is crucial for scenarios where understanding the dialogue structure and dynamics between multiple speakers (such as an examiner and a patient) is essential. Our choice of Google’s technology for speaker diarization and transcription was driven by its superior capability to recognize and label speakers based on the diagnosis scene contextually. Unlike other technologies, such as Microsoft’s, which generally label speakers numerically (e.g., Speaker 1, Speaker 2) without any context of their roles, Google’s tools can accurately identify and distinguish roles (e.g., Examiner, Patient) within the conversation. This functionality is crucial for our assessments as it allows for a precise analysis of the dialogues in terms of who is speaking, enhancing the contextuality and relevance of the linguistic data extracted.

We employed the following steps: 1. Audio Segmentation: Each audio recording, typically longer than one hour per subject, is segmented based on the scenario (e.g., describing a picture, interpreting cartoons). This targeted segmentation helps isolate the conversations relevant to specific assessment tasks, making them more manageable and focused for analysis. 2. Model Configuration: The Medical-Conversation model setting is used to recognize and differentiate between two primary speakers, typically the ‘Examiner’ and the ‘Patient’, within a medical or diagnostic context. By configuring the transcription service to recognize these roles specifically, we ensure that the transcription outputs are accurately labeled, reflecting the dialogue true dynamic. 3. Speaker Diarization and Speech-to-Text Transcription: Applying Google’s diarization algorithm, each segmented audio file is processed to identify and label the speakers’ voices throughout the conversation. Following the diarization, the speech-to-text transcription is executed on these segmented files. This process guarantees that each speaker’s contributions are correctly identified and transcribed, providing a reliable textual basis for subsequent linguistic analysis.

### 4.4 Diagnosing Language Disorders Associated with Autism via ChatGPT

This subsection details the methodology used to employ ChatGPT, an advanced language model, for diagnosing Social Language Disorders (SLDs) in individuals with ASD. The approach leverages a structured prompt to analyze dialogues between examiners and patients, determining the presence of communicative impairments characteristic of ASD.

#### Prompt Design for ChatGPT:

This process involved parts. In Part 1, Examiner-Patient Dialogue (EPD), the input to ChatGPT included the transcribed dialogue between the examiner and the patient, presenting the conversational context needed for assessment. In Part 2, Question (Q), following the dialogue, ChatGPT was asked: ”Based on the above conversation between the examiner and the patient, please categorize if any observed SLDs for the patient. Answer only ‘Yes’ or ‘No’.” This question aimed to elicit a definitive response based on the dialogue’s content, focusing solely on the presence or absence of disorder indicators.

#### Response Interpretation Using ChatGPT:

The responses from ChatGPT were parsed to determine the presence of SLDs. The decision process was as follows:

- **Response Parser**: Each response from ChatGPT, indicating either affirmation (”Yes”) or negation (”No”), was analyzed to ascertain whether the patient exhibited symptoms of SLDs based on the dialogue provided. The parser specifically looked for expressions of affirmation or negation concerning the presence of communicative impairments.
- **Diagnosis Determination**: For each subject, a diagnosis of a SLD was considered positive if there was at least one scenario where ChatGPT affirmed the presence of SLDs (”Yes”). Conversely, if all scenarios resulted in a ”No” from ChatGPT, the subject was not considered to have SLDs as per the dialogues analyzed.

#### Significance of This Approach:

Utilizing ChatGPT for this purpose offers several advantages:

- **Scalability**: The ability to process large volumes of dialogue data quickly and uniformly without human interviewer bias.
- **Consistency**: Standardized prompt responses ensure that the assessment criteria remain constant across all examinations.
- **Precision**: Advanced natural language understanding allows ChatGPT to detect subtle nuances in dialogue that may indicate disorders, which are often challenging for human evaluators to perceive consistently.

By integrating ChatGPT’s advanced analytical capabilities, this methodology refines the diagnostic process for social language disorders in ASD, enhancing both the efficiency and accuracy of assessments. This approach not only supports clinicians by providing a reliable diagnostic tool but also contributes to the broader field of psycholinguistics by offering insights into the communicative impairments often seen in ASD.

### 4.5 Identifying Language Disorder Features Associated with ASD via ChatGPT

This subsection elaborates on the methodology employed to harness ChatGPT for identifying specific language disorder features associated with ASD, guided by expert knowledge integrated from the ADOS-2, Module 4. The approach utilizes a comprehensive list of language disorders designed around the nuanced communication requirements and symptoms observed in verbally fluent adolescents and adults.

#### 4.5.1 Autism-Associated Language Disorders features

Based on the domain knowledge of professional ADOS-2 examiners, ten specific features of language deficits related to ASD have been identified. These features reflect various unconventional use patterns of language that can signify underlying social communication issues. Table 8 is a detailed description of these features:

#### 4.5.2 ChatGPT Prompt Design for Feature Extraction

To facilitate the extraction of these features of language deficits using ChatGPT, a specific prompt structure is utilized, as shown in ‘Prompt 2’ (Figure 4). The prompt was organized into three parts to optimize the analysis, including Part 1: Examiner-Patient Dialogue (EPD), Part 2: Question (Q) - …, and Part 3: Knowledge (K) - ”Overview of the 10 features of social language disorders identified by ADOS-2 examiners, as shown in the column ‘Explanation’ in Table 8

This structured prompt design guides ChatGPT to analyze the transcribed conversations and categorize language deficits, enhancing the precision of diagnostics based on observed linguistic patterns.

##### Response Interpretation Using ChatGPT:

ChatGPT’s responses were analyzed to determine the presence and types of SLD features as follows:

- **Response Parser**: The parser reviewed ChatGPT’s responses, which involved multiple labels corresponding to the 10 predefined SLD features. Each piece of dialogue could yield several labels, reflecting the multi-dimensional nature of language disorders.
- **Feature Classification**: Each response was predicted into multiple categories, constituting a multi-label classification task. This approach allowed for a comprehensive assessment of the patient’s language abilities, identifying multiple SLD features from a single excerpt of dialogue.

##### Significance of This Multi-Label Classification Approach:

- **Comprehensive Analysis**: By classifying dialogue into multiple SLD categories, ChatGPT provides a nuanced view of the patient’s communicative impairments, offering detailed insights that are critical for accurate diagnosis.
- **Targeted Interventions**: Identifying specific disorder features allows clinicians to design more focused and effective intervention strategies, tailored to address the distinct challenges faced by the patient.

Utilizing ChatGPT to identify and classify language disorder features via a structured multi-label classification approach significantly refines the diagnostic capabilities in ASD assessments. This methodology not only enhances the accuracy of the diagnoses but also deepens the understanding of the patient’s specific communicative deficits, facilitating the development of targeted therapeutic strategies.

## 5 Conclusion

This research confirmed the substantial benefits of integrating LLMs such as ChatGPT with the ADOS-2 procedures for diagnosing ASD in adults. Utilizing ChatGPT, enhanced with Google’s speaker diarization and transcription technologies, significantly improved the accuracy, precision, recall, and F1 score of language deficit diagnoses compared to traditional models. This integration not only streamlines the diagnostic process, making it more efficient and less subjective but also enhances the scalability of interventions, providing faster and more accurate assessments that are crucial for effective treatment planning.

Looking forward, the study highlights the potential for these technologies to incorporate a wider variety of data and to develop adaptive learning models that continually improve in accuracy and effectiveness. This progression promises to revolutionize ASD diagnostics, paving the way for more personalized and accessible care for individuals with ASD. The integration of LLMs like ChatGPT in clinical settings is a forward step in making ASD diagnostics not only quicker and more accurate but also more comprehensive in understanding and addressing the diverse needs of the autism community.

## Figures and Tables

**Table 1:** Performance Metrics of Different Models

Model	Accuracy	Precision	Recall	F1 Score
XLNet [20]	58.76%	54.38%	58.76%	56.07%
ALBERT [21]	69.07%	47.71%	69.07%	56.44%
DistilBERT [22]	58.76%	51.85%	58.76%	54.44%
RoBERTa [23]	57.73%	58.12%	57.73%	57.92%
BERT [15]	63.92%	60.86%	63.92%	61.87%
**ChatGPT-based (Ours)**	**81.82%**	**82.45%**	**81.82%**	**79.89%**

**Table 2:** Ablation Experiments: Performance Metrics of Different Schemes

Model	Accuracy	Precision	Recall	F1 Score
wo/ Speaker Diarization	63.64%	48.12%	63.64%	54.80%
w/ Pyannote	68.18%	59.68%	68.18%	60.45%
w/ Microsoft	72.73%	71.25%	72.73%	66.10%
**w/ Google**	**81.82%**	**82.45%**	**81.82%**	**79.89%**

**Table 3:** Prevalence of Linguistic Features by Scenario Indicative of Language Deficits in ASD

Scenario	3	4	5	6	7	9	11	12	13	14	15
*F*_1_	0.87	0.00	0.00	0.00	0.00	0.77	0.00	0.00	0.00	0.00	0.63
*F*_2_	0.90	0.81	0.81	0.87	0.84	0.81	0.77	0.94	0.77	0.80	0.90
*F*_3_	0.81	0.74	0.81	0.90	0.77	0.61	0.77	0.81	0.53	0.67	0.50
*F*_4_	0.68	0.00	0.00	0.00	0.00	0.65	0.00	0.00	0.00	0.00	0.50
*F*_5_	0.61	0.00	0.00	0.00	0.00	0.65	0.00	0.00	0.00	0.00	0.67
*F*_6_	0.94	0.90	0.74	0.90	0.81	0.77	0.81	0.84	0.83	0.87	0.93
*F*_7_	0.77	0.77	0.87	0.87	0.97	0.74	0.90	0.90	0.80	0.63	0.70
*F*_8_	0.71	0.65	0.65	0.74	0.77	0.48	0.68	0.74	0.63	0.50	0.57
*F*_9_	0.61	0.00	0.00	0.00	0.00	0.42	0.00	0.00	0.00	0.00	0.43
*F*_10_	0.71	0.77	0.74	0.81	0.90	0.42	0.81	0.90	0.63	0.57	0.50

**Table 4:** Case Study Analysis: Identifying Language Deficits in an Examiner-Patient Dialogue. Phrases highlighted in blue indicate observed linguistic anomalies, while red underscores the specific feature category of language deficits.

Examiner (E) - Patient(P) Dialogue
E: Okay. So, do you have some friends?
**P: Uh, do I have some friends?*F***_**1**_
E: Um-hum.
**P: Well, pretty much*F***_**10**_ **, three of them from peers.**
E: Three of them from here?
**P: Um-hum.**
E: Okay. Can you tell me a little bit about them?
**P: Well, they’re kind of living near, they kind of live near her*F***_**3**_ **farther from here.**
E: They’re further from here? What do they like?
**P: What do they like?*F***_**1**_ **They’re kind of energetic, just like me. Cool.**
E: Um, and what do you guys like to do together? Man, we like movies and stuff.
P: And you’ve got to know them through peers.
…
E: And, but you said you’d go, you’d like to go to movies and stuff as well. Do you go to movies with them, or?
**P: We use gas movies*F***_**9**_.
E: Oh, you talk about it?
**P: Yeah.**
E: Okay. And are there people outside of peers that you’re friends with, or?
**P: You mean, uh, outside of peers*F***_**1**_ ?
…
**P: Crazy, crowded, crooked.. One of those years people.**
E: Oh.
**P: They triggered the trip, pregnant sound effects.*F***_**9**_
E: Oh, yeah.
**P: When they asked about dating.. Um What, where do you, uh, want to live when you get older**
E: face?
**P: I want to live in a lounge and dirty autistic matching *F***_**2**_**. You know, you can zillion blocks matching*F***_**10**_.
E: And who do you think you would like to live with, with your family or roommates or by yourself?
**P: I want to live with my family there. Okay**
…
Extracted Features Based on ChatGPT Response
**Echoic Repetition** (*F*_1_): When the examiner first asked the patient whether they have some friends, the patient echoed the question back at the examiner before answering. In subsequent interactions, the patient frequently mimics the examiner’s questions verbatim before answering.
**Unconventional Content** (*F*_2_): The patient refers to living in ”a lounge and dirty autistic matching” rather than using any conventional descriptions for living spaces. Similarly, the phrase ”zillion blocks matching” has an unusual content.
**Pronoun Displacement** (*F*_3_): The patient referred to his own house as ”her”.
**Stereotyped Media Quoting** (*F*_9_): The patient quoted ”gas movies” and “triggered the trip, pregnant sound effects”, which seems to be quoted from an external media source.
**Clichéd Verbal Substitutions** (*F*_10_): The patient uses clichéd expressions like ”well, pretty much” instead of giving direct responses.

**Table 5:** Case Study Analysis: Identifying Language Deficits in an Examiner-Patient Dialogue. Phrases highlighted in blue indicate observed linguistic anomalies, while red underscores the specific feature category of language deficits.

Examiner (E) - Patient(P) Dialogue
E: So, I’m going to ask you a few questions about work and school
**P: Yes.**
E: Um, first of all, do you have a job?
**P: No, I used to be laid off.**
…
E: And that’s okay? Yeah Um, while you were working or now at school, or at high school, maybe before that, did you have a group of, any problems getting along with people You weren’t in high school?
**P: Any school. Well, like, like, stupid schools for you when I was developing angry or high school.*F***_**2**_
…
E: What kind of things you used to bother you that other people did?
**P: Like, uh, when I was in the school bus I had students grabbing my backpack, whatever, and I didn’t mad it or suck.*F***_**10**_
…
E: And have you ever done anything so that other people wouldn’t teach soon?
**P: Yes, but sometimes they just, it’s like they’ve been doing it for a while, so it’s just kind of like Hey, you know or what, whatever,*F***_**6**_ **we’ll just tease him about something else.**
…
Extracted Features Based on ChatGPT Response
**Unconventional Content** (*F*_2_): There are instances where the patient uses unconventionally chosen phrases like ”stupid schools for you when I was developing angry or high school”.
**Superfluous Phrase Attachment** (*F*_6_): The patient attaches redundant phrases or filler expressions to their speech without contributing any substantive meaning or context, such as ‘whatever’ and ‘or whatever’.
**Clichéd Verbal Substitutions** (*F*_10_): The patient resorts to clichéd expressions when describing how he felt during certain situations: ”I didn’t mad it or suck.

**Table 6:** Overview of Scenario Tasks in ADOS-2 Module 4 Diagnosing process

Scenario	Name	Explanation
*S*_1_	Construction Task	Involves the participant engaging in a task that requires constructing or assembling a set structure, testing spatial and motor skills rather than communicative abilities.
*S*_2_	Telling a Story from a Book	Primarily a monologic task where the participant recounts a story from a book, differing from spontaneous dialogic interactions.
*S*_3_	Description of a Picture	Participants describe a picture, testing their ability to interpret visual information and articulate a coherent description.
*S*_4_	Conversation and Reporting	Focuses on the ability to engage in back-and-forth conversation and to report on past events.
*S*_5_	Current Work and School	Discusses participants’ current educational and occupational engagements.
*S*_6_	Social Difficulties and Annoyance	Elicits experiences of social challenges and annoyances.
*S*_7_	Emotions	Requires participants to express and identify emotions.
*S*_8_	Demonstration Task	Requires the participant to demonstrate how to use an item or explain a process, which does not involve interactive communication with an examiner.
*S*_9_	Cartoons	Involves interpreting sequences and explaining cartoon strips.
*S*_10_	Break	A pause or intermission in the assessment, involving no communicative or cognitive tasks.
*S*_11_	Daily Living	Covers daily routines and personal care tasks.
*S*_12_	Friends, Relationships, and Marriage	Discusses personal relationships and social norms regarding friendships and marital status.
*S*_13_	Loneliness	Addresses feelings and situations of loneliness and isolation.
*S*_14_	Plans and Hopes	Involves discussing future aspirations and plans.
*S*_15_	Creating a Story	Tests creative storytelling abilities in an unstructured task.

**Table 7:** Detailed Assessment Categories for the ADOS-2 Module 4 Observations

Class	Name	Items	Description
A	Language and Communication	A1 ~ A10	Assesses the ability to use speech and gestures in communication effectively, evaluating the clarity, coherence, and appropriateness of language used in social interactions.
B	Reciprocal Social Interaction	B1 ~ B13	Focuses on non-verbal and verbal behaviors used in social interactions, including eye contact, facial expressions, body postures, and the quality of speech interactions.
C	Imagination / Creativity	C1	Evaluates the subject’s ability to use imagination and creativity in their expressions and thoughts, such as storytelling or creating novel responses to social scenarios.
D	Stereotyped Behaviors and Restricted Interests	D1 ~ D5	Includes specific behaviors that are repetitive, restricted, and stereotyped. This category assesses the frequency and intensity of these behaviors as indicators of ASD.
E	Other Abnormal Behaviors	E1 ~ E3	Observes behaviors that are typically considered abnormal, such as overactivity, anxiety, and emotional responses that are inconsistent with the normative context.

**Table 8:** Descriptive Analysis of Unconventional Language Disorder Patterns

F	Name	Explanation
*F*_1_	Echoic Repetition	The individual mimics verbatim what has been said by others, including the examiner, or recites phrases from external sources like advertisements or movie scripts, showing a delayed echo response.
*F*_2_	Unconventional Content	The speech contains peculiarly chosen content or contextually odd phrasing, such as using ‘unfreshness through household’ for lack of novelty, ‘mideast’ instead of ‘midwest’ for U.S. states, or describing entry into a building as ‘through various apertures’.
*F*_3_	Pronoun Displacement	Incorrectly substitutes personal pronouns, using ‘you’ in place of ‘I’, or refers to themselves in the third person, either by pronouns like ‘he/she’ or by their own name.
*F*_4_	Incongruous Humor Timing	Incorporates humorous or comedic expressions inappropriately during discussions meant to be serious, showing a misalignment between the content’s emotional tone and the context.
*F*_5_	Formalistic Language Use	Employs an overly formal or archaic language style that seems lifted from written texts, legal documents, or old literature, rather than engaging in conversational speech. Examples include elaborate ways of expressing simple ideas or feelings.
*F*_6_	Superfluous Phrase Attachment	Attaches redundant phrases or filler expressions to their speech without contributing any substantive meaning or context, such as ‘you know what I mean’ or ‘as they say,’ indicating a habit rather than intentional emphasis.
*F*_7_	Excessive Social Phrasing	Utilizes conventional social expressions excessively or inappropriately, responding with phrases like ‘oh, thank you’ in contexts where it does not fit or preempting social gestures not yet performed by the interlocutor.
*F*_8_	Monotone Social Expression	Reiterates social phrases with an unchanged, monotonous intonation, indicating a lack of genuine emotional engagement or variability in social interactions.
*F*_9_	Stereotyped Media Quoting	Quotes lines from commercials, movies, or TV shows in a highly stereotypical manner, employing a canned intonation that mimics the original source closely, suggesting a reliance on external media for verbal expressions.
*F*_10_	Clichéd Verbal Substitutions	Resorts to well-known sayings or clichés in lieu of engaging in direct conversational responses, using phrases like ‘circle of life’ or ‘ready to roll’ as stand-ins for more personalized communication.
